# Current state and future prospects of pure mycelium materials

**DOI:** 10.1186/s40694-021-00128-1

**Published:** 2021-12-20

**Authors:** Simon Vandelook, Elise Elsacker, Aurélie Van Wylick, Lars De Laet, Eveline Peeters

**Affiliations:** 1 Research Group of Microbiology, Department of Bioengineering Sciences, Vrije Universiteit Brussel, Pleinlaan 2, B-1050 Brussels, Belgium; 2 Research Group of Architectural Engineering, Department of Architectural Engineering, Vrije Universiteit Brussel, Pleinlaan 2, B-1050 Brussels, Belgium; 3 Hub for Biotechnology in the Built Environment, Devonshire Building, Newcastle University, Newcastle upon Tyne, NE1 7RU UK

**Keywords:** Pure mycelium materials, Biomaterials, Myco-leather, Mycelium foam, Fungal leather, Aerial hyphae

## Abstract

In the context of the ongoing transition from a linear to a circular economy, ecologically friendly renewable solutions are put in place. Filamentous fungi can be grown on various organic feedstocks and functionalized into a range of diverse material types which are biobased and thus more sustainable in terms of their production, use and recycling. Pure mycelium materials, consisting only of mycelial biomass, can adopt versatile properties and appear promising as a substitute for current petrochemically produced polymeric materials or, in the case of myco-leather, as a substitute for animal-based leather. In recent years, a handful of private companies have been innovating to bring products based on pure mycelium materials to the market while scientific interest in these promising biomaterials is now starting to gain momentum. In this primer, we introduce pure mycelium materials, frame different production methods, review existing and potential future applications, thereby offering a vision on future advances for this emerging fungi-based technology.

**Supplementary Information:**

The online version contains supplementary material available at 10.1186/s40694-021-00128-1.

## What are (pure) mycelium materials?

**Fig. 1:**
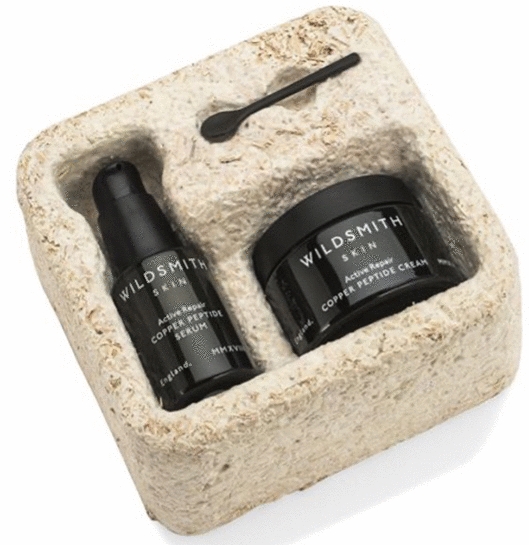
Composite mycelium material used as packaging alternative to expanded polystyrene ©Ecovative

Filamentous fungi display an intrinsic growth pattern that generates a near endless amount of microscopically interconnected tubular cells eventually yielding a vast macroscopic network of biomass, also known as mycelium. In nature, these heterotrophic organisms fulfil key ecological roles by breaking down and recycling diverse sources of biomass, in terrestrial and marine habitats, thanks to their powerful secretion abilities exploited in many biotechnological applications [1]. In the last decades, this bioconversion process has been introduced into an anthropogenic context, resulting in a biobased production process of new types of natural materials with highly tuneable properties [2–5]. Clear signs of innovations and a rising trend in patent applications indicate that the use of fungal mycelium as a raw resource will help in providing a scalable solution for sustainable material alternatives [6]. Initially, focus was mainly placed on the development of composite mycelium materials (CMM). By inoculating lignocellulosic waste streams from agricultural or forestry origin (*e.g.* corn husks or sawdust) with a fungal saprotrophic species, typically belonging to white-rot genera (*e.g. Ganoderma* or *Pleurotus*), inside a mould, colonization of the organic feedstock occurs [7]. The expanding fungal filaments bind substrate particles together while filling in void spaces and ultimately a composite material shaped by the mould is formed, which is subsequently processed by drying thereby ending the growth process by dehydrating the organism [7, 8]. In essence, the feeding fungus acts as a biological glue for the material by colonizing and binding the loose substrate particles. The resulting lightweight CMM have foam-like material characteristics (*e.g.* polystyrene), thereby being suited for applications such as packaging, insulation and lightweight furniture applications (Fig. 1) [9–13].

**Fig. 2:**
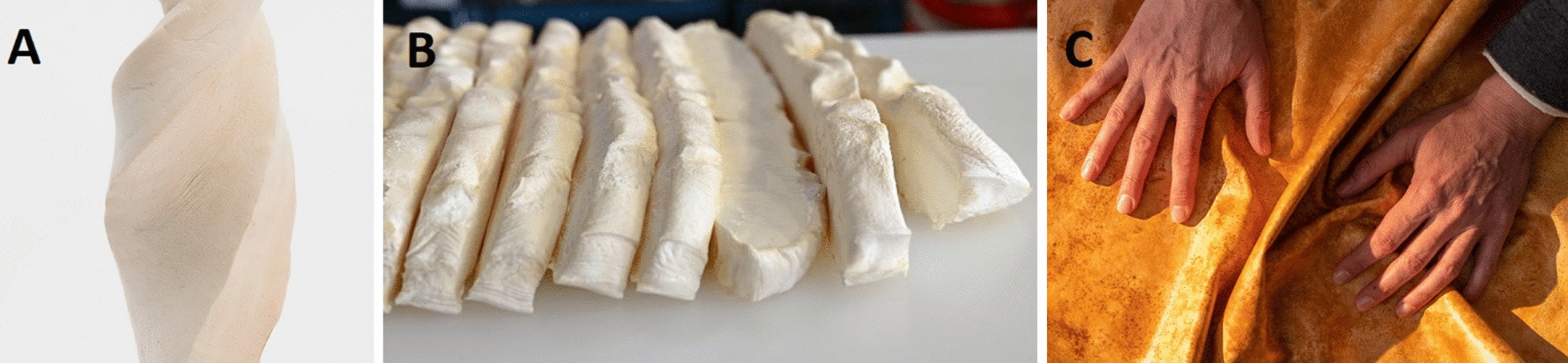
Pure mycelium products: **A**. Mycoflex™ is a foam like material with resilient properties, **B**. Atlast™ is a mushroom based meat alternative, **C**. Reishi™ is a mycelium-based leather alternative to animal skins and plastic leathers

In contrast to CMM, in which the lignocellulosic substrate is intrinsic to the composition and characteristics of the material, the authors define pure mycelium materials (PMM) as to not contain left-over feedstock particles, so the basic properties of PMM are fully determined by the biological characteristics of the organism and growth conditions. Pure mycelium biomass can be generated by growing the organism in separation of its substrate to yield sustainable materials with tuneable properties ranging from foam-, paper-, leather- to polymer-like characteristics. Additionally, newly developed fermentation processes for PMM further increases possibilities to functionalize this unique material, when combined with the appropriate post-growth processing treatment of the mycelium [14–17]. As the range of properties for PMM expands, a growing number of consumer products from companies ensue ranging from high-performance foams used in apparel or skincare products (Mycoflex™) [18] to leather like fabrics (Mylo™, Reishi™, Mylea™, Forager™) to meat alternative products (Atlast™) (Fig. 2) (Additional file 1: Table S1).

Mycelium products have a major advantage with respect to their synthetic counterparts in single use applications in terms of carbon footprint and sustainability. When mycelium materials, encompassing both CMM and PMM, are not treated for long term preservation or not combined with non-biodegradable elements, they are a great solution for short carbon turnover and recyclability [19]. Additionally, mycelium materials were shown to have flame-retardant properties and are less likely to combust as compared to petrochemical-derived plastics [19–21]. Finally, the growth phase of mycelium materials is a relatively fast process that can be achieved in 5 to 14 days when using an efficient fermentation setup, depending on fungal species and fermentation conditions [14, 22, 23].

PMM represent a promising novel technology, not only because of the versatility of novel material applications but also because of a possible feedstock diversification. Indeed, they can be produced through fermentation processes in which low value agricultural by-products can be upcycled as a nutritive vehicle to promote growth of fungal biomass with the possibility of integrating one or more additional specie(s) in a co-cultivation setup [24]. In this primer, we will discuss the most prominent advances in research and development of PMM and highlight future opportunities.

## Three advances of research in the last decade

### Fermentation strategies to produce pure mycelium materials

**Fig. 3:**
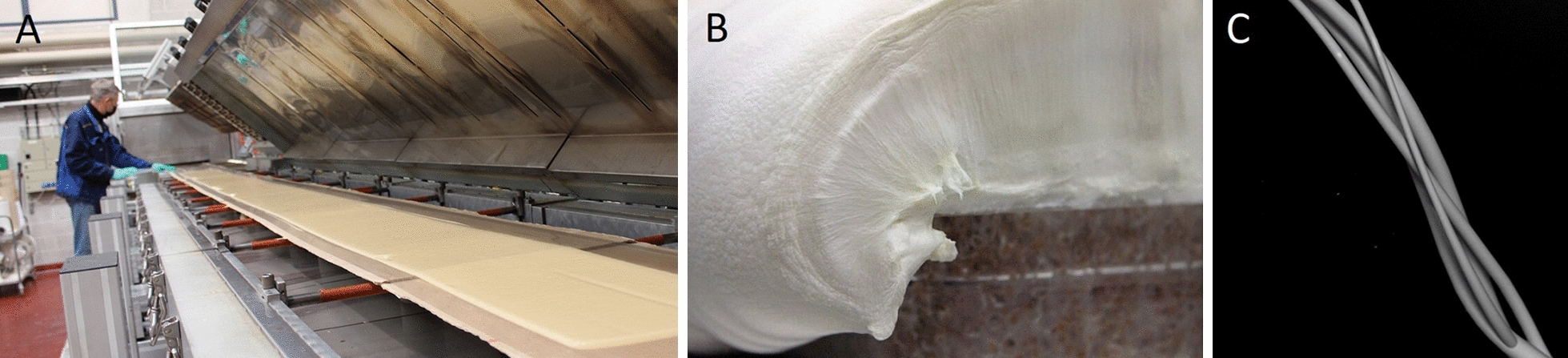
**A**. Pilot scale of continuous mycelium leather production at VTT bioreactor fermentation [86]., **B**. Outward growth of aerial hyphae in solid state fermentation setup ©Ecovative, **C**. Virtual animation of the microscopic arrangement of hyphae into an interlocking cellular structure named Fine Mycelium™ [87]

In contrast to CMM, for which a customized but relatively uniform production process has been established, PMM can be produced using diverse fermentation technologies. In general, fungi require direct contact with their nutrient source hence, this is why they grow intimately bound to their feedstock. Therefore, liquid fermentation has for a long time been the technique of choice when needing to recover metabolites, enzymes or mycelial biomass. Consequently, bioreactor fermentation is a mature and established technology that has been scaled to large industrial volumes [25]. Besides, this way of cultivating fungal mycelium is well-studied and enables to control every process parameter. For this reason, bioreactor fermentation set-up appears very attractive to produce PMM. Recently, scientists at VTT Technical Research Centre of Finland demonstrated continuous mycelium leather production using mycelium from submerged bioreactor fermentation (Fig. 3A) [22].

Growth of pure mycelial biomass can also be achieved on solid feedstocks. Recent innovations in solid state fermentation (SSF) have determined specific growth conditions that stimulate abundant growth of aerial hyphae as shown for *Ganoderma sp.* (Fig. 3B) [14, 26–28, 31]. This type of hyphae is characterised by an outwards growth, away from the substrate, into the air [29]. To induce this growth behaviour, temperature (± 30 °C), gaseous CO_2_ (50–70 k ppm) and relative humidity (40–99%) are tightly controlled inside incubation chambers [14, 26–28]. These conditions are optimal to prevent any differentiation of the mycelium into fruiting bodies since mushroom growers use lower temperature and CO_2_ concentrations for optimal fruiting yields [30]. To our knowledge, there are no detailed reports available on the detailed growth modelling for this new fermentation strategy with respect to fermentation parameters or to specific species or genera used. From the limited available literature, members of the Polyporales such as *Ganoderma sp.* appear suited to grow mycelium foams [31]. According to Greetham et al. [14] the main driving force stimulating the outwards growth of the mycelium, in the above-mentioned conditions, is generated by the carbon dioxide gradient induced through cellular respiration. The build-up of CO_2_ inside the substrate vehicle naturally creates a gradient alongside which the mycelium will grow outwards to reach a more hospitable environment [14, 26]. A second driving force stimulating vertical expansion results from generating an osmotic potential at the apex. Through the deposition of solute-containing microdroplets (solute being a mineral, protein or carbohydrate), hyphal extension, which is driven by turgor pressure, can be steered and stimulated [26, 32]. Furthermore, applying a constant flow of air above the growing mycelium, modulates the outwards growth and shape of the biopolymer [26]. As reported by Kaplan-Bie et al. [26], the rate at which air is displaced above the substrate boxes combined with relative humidity levels directly impacts the density of the aerial mycelium and modulates the degree of homogeneity from the resulting mycelium foams (Table 1).

**Table 1:** Influence of varying airflow rates and relative humidities on the dry density and tensile strength of mycological biopolymers from *Ganoderma sp*. at 5% CO_2_ and ± 30 °C in a customized incubator [26, 31]

Airflow rate (m^3^/minute)	Relative humidity	Dry density (kg/m^3^)	Tensile strength (MPa)
2,83	> 99%	31,72	0,12
2,83—4,95	> 99% drop to 96% for 48 h	23,23	0,09
8,49—10,62	> 99%	53,18	0,21

### Processing and functionalization of mycelium

When considering the further processing of PMM, previously established post-growth processing steps of CMM can be inspirational. Lignocellulosic CMM are appealing for applications for which a rigid material is desired (hard packaging, insulation, furniture). In this instance, physical treatments through heat and/or compression are suited to dehydrate the mycelium resulting in an increased rigidity while preventing further growth. Heat pressing the material provides an increased density while promoting heat-induced crosslinking of molecular bonds [7, 8].

**Fig. 4:**
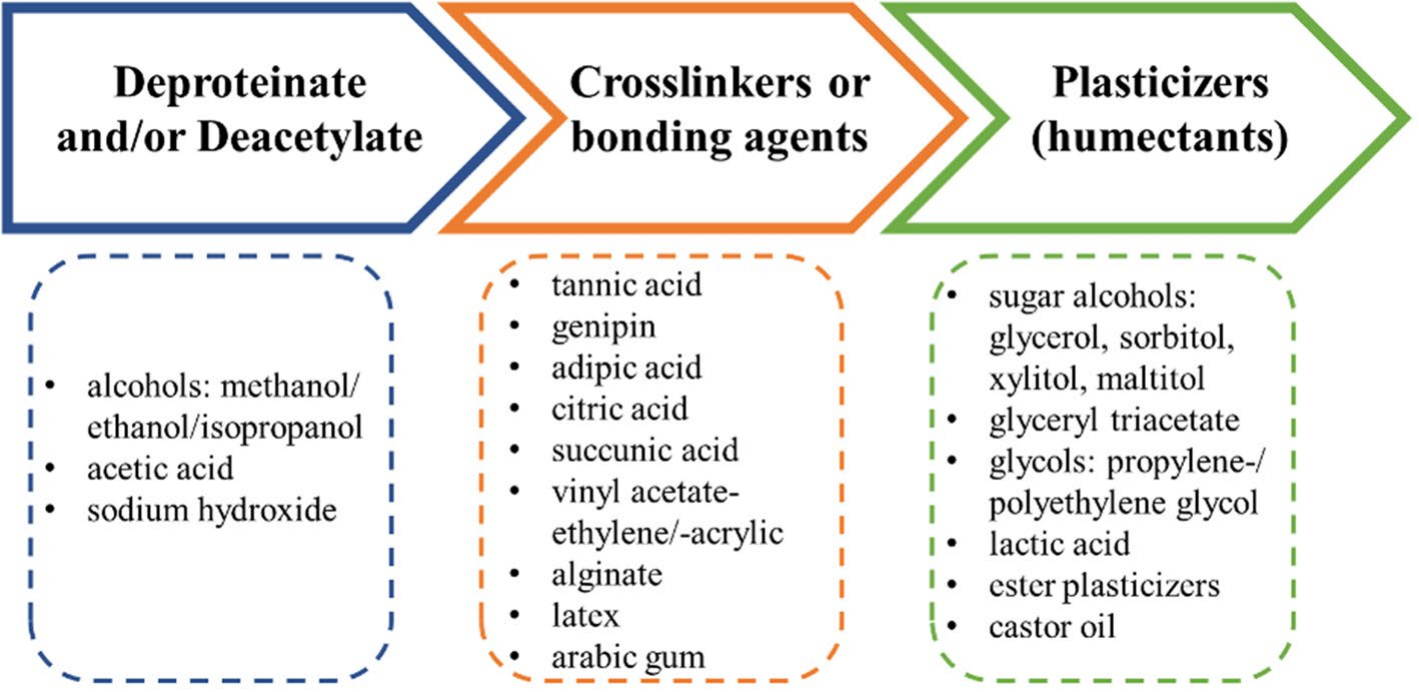
Compounds used in the treatment of mycological tissue for material applications [16, 17, 22, 35, 36, 41, 46, 88]

In contrast, for PMM, flexibility is preferred over rigidity for applications such as fabrics and flexible foams [33]. For this, different chemical treatments can be applied to the fungal tissue (Fig. 4) and also provide pliable or absorbent properties, durability and a protective coating [16, 17, 22, 34–36]. So far it is unclear which exact formulation of post-growth processing elements and steps yield the best results as the combinations are numerous and this research field is ongoing, but we tried to regroup the most relevant steps and elements in Fig. 4 to provide a simplified general overview.

**Fig. 5:**
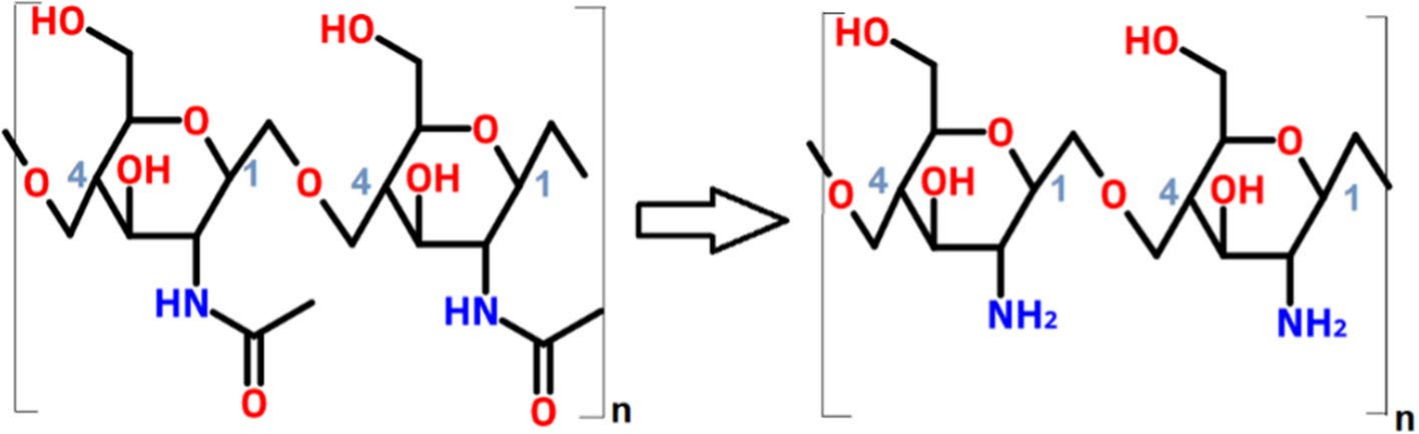
Deacetylation of chitin (N-acetyl-D-glucosamine) to chitosan (D-glucosamine)

The fungal cell wall contains the prevailing building blocks that physically characterize products made from pure mycelium. These building blocks are glucans, chitin/chitosan and glycoproteins, which can vary in their compositional ratio depending on the species, environmental conditions and developmental stage [37–39]. As such, it provides an attractive platform for the introduction of chemical modifications thereby enabling new properties. Chitin, which is the second most abundant polysaccharide on earth, refers to a *N*-acetyl-d-glucosamine polymer with an acetylation level > 50% while chitosan terms acetylation levels lower than 50% [40]. Deacetylation of this biopolymer liberates amino (NH_2_) groups that can afterwards be used as additional crosslinking sites on the chitosan backbone structure in a similar manner as the already available hydroxyl (OH) group (Fig. 5). Additionally, CaCl_2_ combined with chitosan has been showed to confer antimicrobial properties and is used in food preservation applications [41]. These antimicrobial properties could find interesting applications in packaging/wrapping applications for products prone to microbial contamination.

PMM are typically coated with the aim to increase longevity and to protect against abrasion. For example, polylactic acid (PLA) offers an effective coating material that can be easily applied when dissolved in water. It is then absorbed by the mycelium and the water evaporates afterwards [35]. Although PLA is considered to be a sustainable bioplastic because it is biosynthesized and can be quickly degraded, the latter is only possible under industrial composting conditions with elevated temperatures (> 60 °C) due to the main depolymerization mechanism being abiotic hydrolysis and not enzymatic degradation [42]. This means that if PLA ends up in the ocean or another natural ecosystem, it most likely won’t have a degradation advantage over other types of plastics [43]. Similarly, cellulose biofilms grown from bacteria (*e.g. Acetobacter xylinus*) are seemingly envisaged as coating material to be applied as a separate coating or in cocultivation with the mycelium [24]. Finally, many other types of coating finishes can be used such as coconut oil, carnauba wax or beeswax [28].

### Myco-leather as a proof-of-concept application

**Fig. 6:**
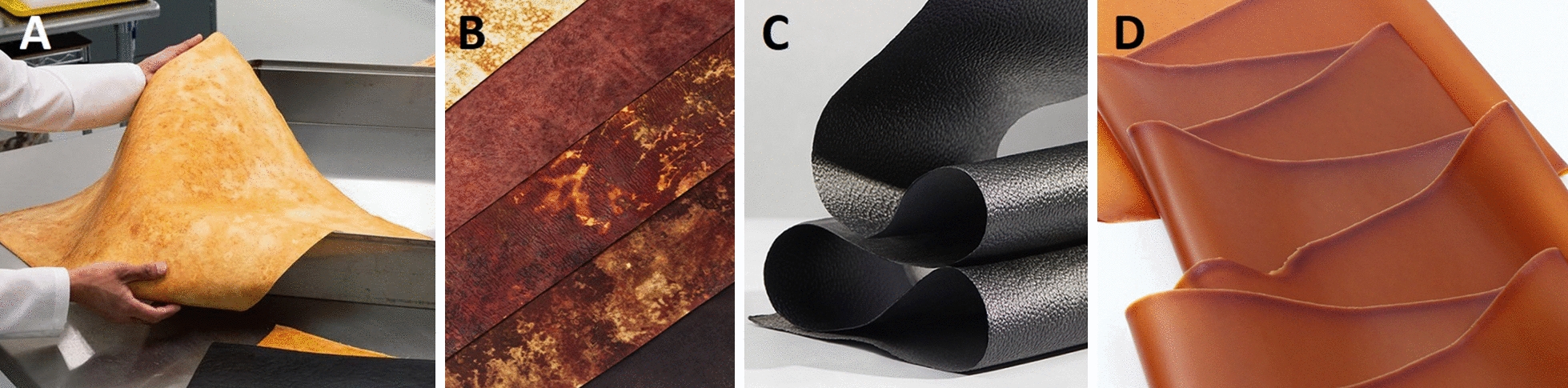
Examples of leather-like mycelium fabrics **A**. Reishi™ from Mycoworks, **B**. Mylea™ from Mycotech Lab, **C**. Mylo™ from Bolt threads, **D**. VTT mycelium leather

The use of fungi as a resource for textile and fabrics is an ancient practice as evidenced by the centuries-old use of traditional German felt (Amadou) and by the identification of mycelium mats in pouches crafted by indigenous North-American people [44, 45]. Moving fast forward into the early development of modern-day PMM, the main targeted application is its use as a leather substitute using species such as *Ganoderma* spp., *Trametes versicolor*, *Fomes* spp., *Pycnoporus *spp. and *Perenniporia* spp. [28, 46, 47]. Traditional leather production is tightly bound to the animal farming industry, which is unequivocally responsible for a significant share of the global greenhouse gas emissions and ever-increasing deforestation [48–50]. While the most common alternatives are synthetic leathers originated from the petrochemical industry, they carry the same environmental burden as non-biodegradable plastics [51, 52]. Myco-leather offers a cleaner alternative and promotes higher sustainability in a sector for dire need of greener improvements [33, 53]. These arguments combined with an annual global market value estimated around USD 394 billion for leather goods in 2020 [54], provide a bright future for commercial success of myco-leather. It is therefore no surprise that companies developing mycelium materials are currently focussing on bringing myco-leather products on the market (Fig. 6).

Variations in mechanical and physical properties between myco-leather brands are noticeable in the reported testing results from the companies (Additional file 1: Figures S1 and S2). To offer a competitive alternative, different strategies can be implemented to enhance the mechanical strength of the material so that it matches that of classical leather. For example, Mylea™ (Fig. 6B) includes a structural fabric-like material that is colonized by the mycelium for improved tear strength properties of the myco-leather product [55]. This fabric can be either natural or synthetic (*e.g.* plant skin, cotton, gauze, grass fibres or artificial polymer fibres) [56]. Such a hybrid material, in which a biobased material is combined with artificial polymers begs questions on the sustainability and biodegradability of the material. As a natural solution, nanocellulose fibrils [57, 58] appear to be a promising biopolymer additive for mycelium leather as they were previously shown to increase tensile strength properties by 271% with a 15% cellulose content and up to 626% for higher cellulose contents in submerged bioreactor fermentation [22].

Bolt Threads’ production process of mycelium leather, based on Ecovative’s fermentation technology, involves compressing a thick mycelium foam (Fig. 3B) into a thin compact tissue [59]. Further downstream treatment of the mycelium, involving bonding agents, ensures sufficient strength to the material in order to fulfil the role of a leather substitute. Interestingly, while Bolt Threads’ Mylo™ product is not plastic free but petroleum free, Ecovative’s Forager™ hides are labelled as 100% plastic free.

Lastly, MycoWorks’ strategy involves arranging the orientation of aerial hyphae to create weaved patterns, similar to regular fabrics, in their Reishi™ product [27, 28]. A recently developed alternative method named Fine Mycelium™ technology, allows them to direct hyphae into a bundled spiral arrangement resulting in highly organized structures similar to rope twisting (Fig. 3C). The company claims that this procedure results in superior mechanical properties with respect to existing mycelium leathers, synthetic leathers and animal leather (Additional file 1: Figure S1). Like traditional textile weaving and lattices, directing changes in fibre orientation creates an overall stronger material composition [60].

## Three areas ripe for development

### Improving production strains

Different fungal species have been successfully demonstrated for their use to grow mycelium in bulk for mycelium material applications. They typically belong to either Basidio- or Ascomycetes although according to the fermentation process and desired application, one might be more suitable than the other. White rot Basidiomycetes belonging to the Agaricales or Polyporales have been reported as suitable candidates to efficiently grow mycelium materials on lignocellulosic substrates (*e.g*. the genera *Ganoderma, Trametes, Pleurotus*, *Fomes* and *Schizophyllum*) [7]. In contrast, when cultivated in bioreactor setups, Ascomycetes (*e.g. Penicillium*, *Aspergillus* and *Trichoderma*) are widely used for biotechnological applications [61]. Besides differences in growth behaviour in different fermentation setups, other valuable traits can provide some significant advantages, such as for example the capability of producing chlamydospores during vegetative mycelium growth. This perennate survival structure enables asexual spore formation, independently from fruiting bodies, which avoids reshuffling of the genetic legacy and is therefore ideal as inoculum source to conserve strain-specific features [62]. An additional aspect to keep in mind when selecting a strain, is the specific cell wall composition. For example, species can vary in the compositional ratio of the chitin/protein content of their cell wall [39], which in turn affects post-processing efficiency (*e.g.* amount of available chemical crosslinking sites) and thus material properties. Taken together, it is important to select the most suited species according to the substrate, fermentation setup and desired material application by screening the existing fungal biodiversity before considering further strain improvements.

In a second instance, strain improvements can be achieved through genetic modifications, which has already been applied for many biotechnological applications [63, 64]. A recent leap forward in targeted genetic modification was made possible with the development of CRISPR/Cas9 as a versatile genetic engineering tool [65, 66]. CRISPR/Cas9-based systems have been established for a range of filamentous fungi but with some remaining challenges [67]. An interesting solution allowing to bypass host-specific transcription systems for non-model organisms is the use of in vitro assembled Cas9 ribonucleoprotein (RNP) complexes [68]. These RNPs can be used across diverse species through polyethylene glycol (PEG) -mediated protoplast transformation without the need for species-specific adaptations as is the case with in vivo expression systems requiring validation of promotors, genetic elements and plasmids. However, it should be noted that protoplastation is not straightforward for many species of filamentous fungi and that other transformation methods might have to be considered [67, 68].

The promise of using genetic engineering to tune mycelium materials has been successfully demonstrated for *Schizophyllum commune* [69]. A *S. commune* mutant strain Δsc3, which is unable to express hydrophobin sc3, was shown to result in mycelium materials with increased density and mechanical strength, thereby shifting their classification from the category of natural materials to thermoplastic-like materials. As another example, the introduction of a *S. cerevisiae* CDA1 chitin deacetylase-encoding gene under control of glyceraldehyde-3-phosphate dehydrogenase (GPD) promoter in a mycelium material production strain has given rise to materials with significantly higher compressive modulus (*i.e.* a material’s stress-to-strain ratio) [70]. Similarly, engineered expression of β-1,3-glucan synthases (BGS1 and BGS2) under control of constitutive promoter of GPD yielded transformants with 135–165% higher β-glucan content in *Ganoderma sp.* [70]. Besides fungi, engineering bacterial strains used during co-cultivation can also be greatly beneficial to improve material properties and avoid contaminants. For example, co-cultivation with engineered *Bacillus* strains secreting polygama-glutamic acid as a biofilm resulted in twofold increased elastic modulus or engineering of melanin production conferred higher protection to UV in mycelium materials [70, 71]. Finally, co-cultivation with an engineered *Streptomyces natalensis* strain producing natamycin (an Ascomycete-specific antibiotic) enabled to supress recurring contaminants like *Trichoderma sp.* [70].

### Upscaling towards industrial production

It is now clear that improvements in fermentation and post-growth processing of mycelium materials that were realized during the past decade have been instrumental in showing their potential as a new class of a sustainable material [26, 31, 33, 72, 73]. As for every prototype design, as challenging as it might be, cost-efficient upscaling for large-scale manufacturing of a new technology will come with new challenges and added complexity. Fortunately, industrial-scale fermentation of filamentous fungi is already established in the food and industrial biotechnological industry. According to the fermentation setup, whether this is a submerged bioreactor or solid-state fermentation strategy, one might need more adaptation to the industrial process than the other. Either way, both strategies come with their own characteristics and result in common and unique material application outcomes.

**Fig. 7:**
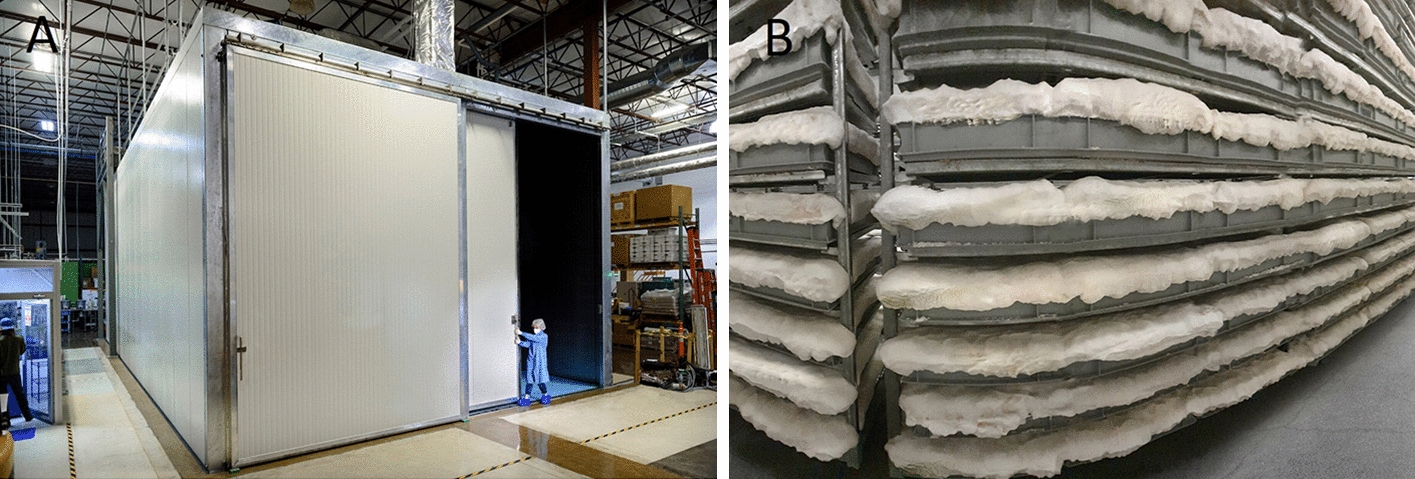
**A**. Incubation chamber for large scale production ©Ecovative, **B**. vertical farming of aerial mycelium foam ©Bolt Threads

Some companies have invested in the development of a large-scale infrastructure for solid-state fermentations. Ecovative showcased their large climate-controlled incubation chamber (Fig. 7A) used to grow substantial quantities of pure mycelium foams at once (Fig. 7B) [26]. Opposed to this, Mycoworks describes their fermentation setup for growing aerial hyphae as consisting of individual boxes, which would be advantageous to minimize the impact of contamination events [27].

Another important factor to keep in mind for industrial-scale production of mycelium materials is the growth substrate. The larger the production plant, the higher the need for substrate will be and if this substrate is not locally sourced, the carbon footprint to produce mycelium materials will increase. Therefore, it is important to identify local sources for mycelium growth substrate and to design the production capacity in function of substrate availability.

### Future applications and transitioning towards a sustainable and circular production model.

So far, Ecovative has demonstrated that lignocellulosic CMM can be a viable alternative to replace expanded polystyrene packaging [74]. Mycelium was also shown to be an interesting support material for electronic circuit boards instead of commonly used acrylic plastics or as e-textiles and reactive fungal wearables [75–77]. Mycelium material properties that are appreciated features for electronic support applications are their heat and thermal resistance, light weight, modulable shape and hydrophobic nature [75].

**Fig. 8:**
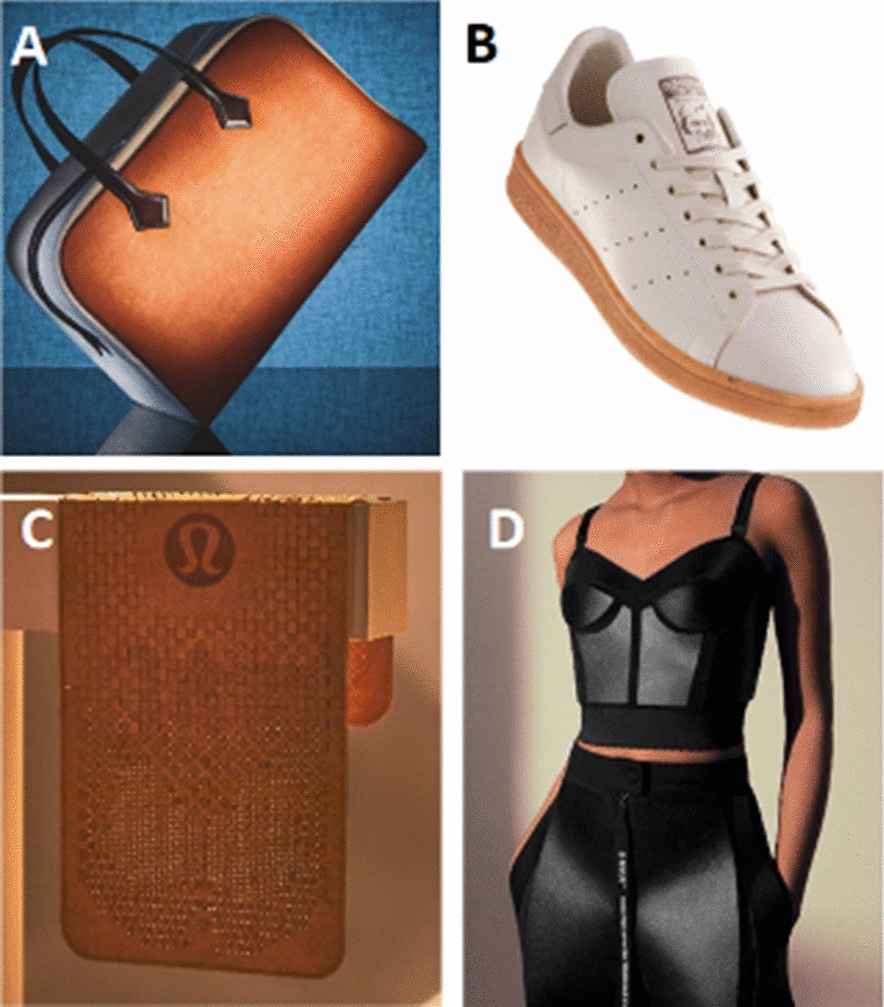
Examples of mycelium-based consumer products by established designer brands: **A** Sylvania by Hermès, **B**. Stan Smith by Adidas, **C**. Yoga mat by Lululemon, **D**. Mylo garment by Stella McCartney.

Recently, attention is shifting towards consumer product applications for PMM. Renowned players in the fashion industry such as Hermès, Stella McCartney, Lululemon or even Adidas have partnered with mycelium leather companies to design prototype consumer products from mycelium leather (Fig. 8). These examples will facilitate the introduction of mycelium materials to a wider range of consumers. In the future, mycelium materials might become more versatile and omnipresent with few noticeable changes. This will not only be stimulated by the expected increase in research and development efforts for this new class of materials, but also because transitioning away from petrol-derived synthetic polymers and animal hides will become an increasing environmental necessity.

Another big impact application is the use of fungi to manufacture meat alternative products. This is not new since Quorn® was the first protein rich meat alternative made from *Fusarium venenatum* going back to 1985 [78], but as reported by the latest Good Food Institute report, many new companies are betting on fermentation technologies and more specifically mycoproteins to further innovate in this space (*e.g.* Nature’s Fynd™, 3F Bio, Atlast Food Co., Chunk, Fybrawork Foods, Kinoko-Tech, Meati, Mycovation, Prime Roots) [79].

Improvements in aerial hyphae fermentation unlocks new structures components that enables to mimic meat texture in a closer way [80]. Additionally, the matrix structure of mycelium materials can be used as wound care material [81, 82] or as a biomedical scaffold to grow biopsies [83]. This self-growing scaffold could serve as a tool to produce lab-grown meat products by culturing myocytes and thereby growing hybrid animal-fungi food products.

Finally, fungal melanin introduces new properties to next-generation engineered melanin-producing mycelium materials as they confer some degree of radioprotection, antioxidant activity to heavy metal chelation and organic compound absorption [84]. It is even considered as a promising option to provide a self-growing radiation shield for humans during future deep-space explorations [85].

## Conclusions

The increasing number of functional applications for mycelium materials qualifies it as a next generation biomaterial even though much of this technology is still in its infancy. So far, major advances in fermentation techniques have resulted in opening new doors for functional applications but many improvements are still required to make it an all-purpose type of material such as plastics. Due to the growth of the organism being highly modulable at the microscopic and macroscopic level, in combination with numerous polymeric or fibre additives and chemical alteration possibilities of cell wall components, mycelium can be referred to as a future platform material with a wide range of tuneable properties. In addition, we strongly believe that the biodegradable characteristics of mycelium materials and circular nature of the production process are the major properties that will further stimulate their breakthrough into consumer markets as a nature friendly product.

## Supplementary Information

**Additional file 1: Table S1.** List of companies working on developing pure mycelium materials that were referenced in this manuscript (table does not provide all existing companies working on myco-leather products). **Figure S1.** Properties of different Reishi^TM^ myco-leather products compared to traditional cowhide leather from MycoWorks [89]. **Figure S2.** Properties of Mylea^TM^ myco-leather from Mycotech lab [55].

## Data Availability

Not applicable.
