# Supplementary material for: Current state and future prospects of pure mycelium materials
Source: Fungal Biol Biotechnol. 2021 Dec 20;8:20. doi: 10.1186/s40694-021-00128-1 (PMC8691024; doi:10.1186/s40694-021-00128-1)

Table S1. List of companies working on developing pure mycelium materials that were referenced in this manuscript (table does not provide all existing companies working on myco-leather products).

| Company | Products | Country | Company size | # mycelium-material related patents |
| --- | --- | --- | --- | --- |
| Ecovative | Atlast, Mycoflex, Mycocomposite, Forager Hides | USA | SME | 43 |
| Mycoworks | Reishi | USA | SME | 11 |
| Bolt Threads | Mylo | USA | SME | 3 |
| Mycotech lab | Mylea | Indonesia | SME | 2 |
| VTT-research institute |  | Finland | N/A | 2 |

Figure S1. Properties of different Reishi^TM^ myco-leather products compared to traditional cowhide leather from MycoWorks.


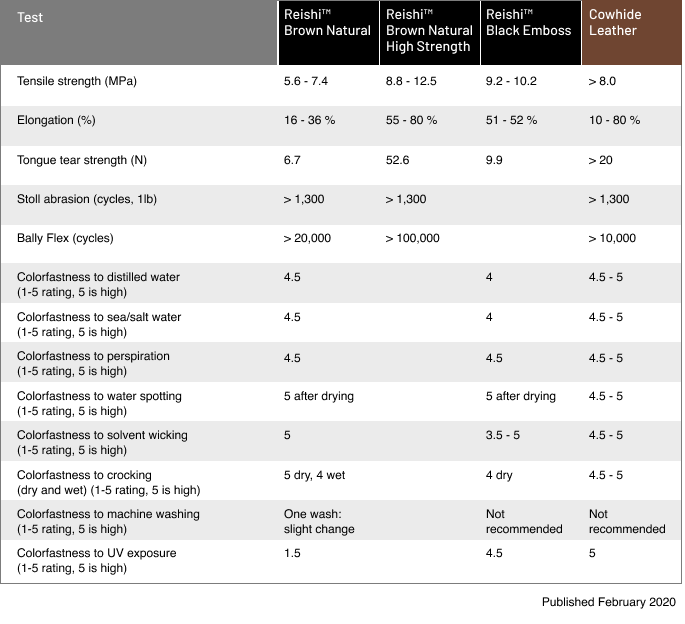


Figure S2. Properties of Mylea^TM^ myco-leather from Mycotech lab (55).


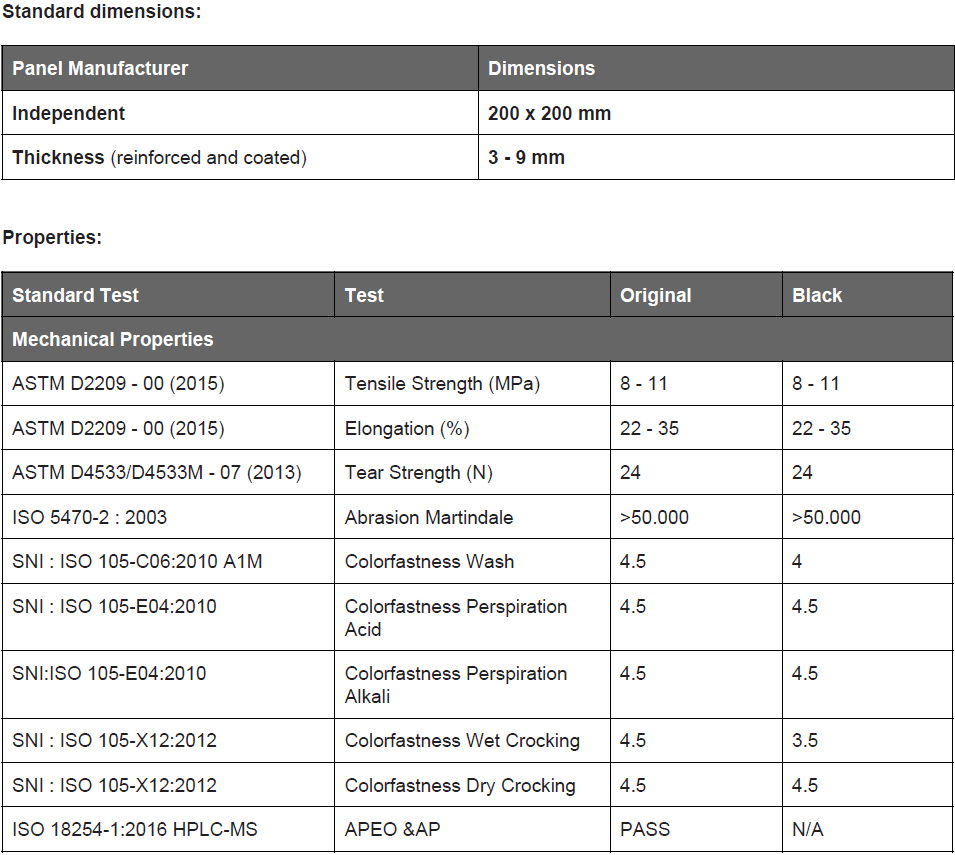

Supplement: Supplementary file 1 — Additional file 1: Table S1. List of companies working on developing pure mycelium materials that were referenced in this manuscript (table does not provide all existing companies working on myco-leather products). Figure S1. Properties of different ReishiTM myco-leather products compared to traditional cowhide leather from MycoWorks [89]. Figure S2. Properties of MyleaTM myco-leather from Mycotech lab [55]. [file 40694_2021_128_MOESM1_ESM.docx]
